# Distinct action of the α-glucosidase inhibitor miglitol on SGLT3, enteroendocrine cells, and GLP1 secretion

**DOI:** 10.1530/JOE-14-0555

**Published:** 2015-03

**Authors:** Eun Young Lee, Shuji Kaneko, Promsuk Jutabha, Xilin Zhang, Susumu Seino, Takahito Jomori, Naohiko Anzai, Takashi Miki

**Affiliations:** 1 Department of Medical Physiology, Graduate School of Medicine, Chiba University, 1-8-1 Inohana, Chuo-ku260-8670, Chiba, Japan; 1 Department of Molecular Pharmacology, Graduate School of Pharmaceutical Sciences, Kyoto University, Kyoto, Japan; 2 Department of Pharmacology and Toxicology, Dokkyo Medical University School of Medicine, Tochigi, 321-0293, Japan; 3 Division of Molecular and Metabolic Medicine, Kobe University Graduate School of Medicine, 7-5-1, Kusunoki-cho, Chuo-ku, Kobe, 650-0017, Japan; 4 Drug Development Center, Sanwa Kagaku Kenkyusho Co., Ltd, 35 Higashisotobori-cho, Higashi-ku, Nagoya, 461-8631, Japan

**Keywords:** miglitol, SGLT3, GLP1 secretion, L cell

## Abstract

Oral ingestion of carbohydrate triggers glucagon-like peptide 1 (GLP1) secretion, but the molecular mechanism remains elusive. By measuring GLP1 concentrations in murine portal vein, we found that the ATP-sensitive K^+^ (K_ATP_) channel is not essential for glucose-induced GLP1 secretion from enteroendocrine L cells, while the sodium-glucose co-transporter 1 (SGLT1) is required, at least in the early phase (5 min) of secretion. By contrast, co-administration of the α-glucosidase inhibitor (α-GI) miglitol plus maltose evoked late-phase secretion in a glucose transporter 2-dependent manner. We found that GLP1 secretion induced by miglitol plus maltose was significantly higher than that by another α-GI, acarbose, plus maltose, despite the fact that acarbose inhibits maltase more potently than miglitol. As miglitol activates SGLT3, we compared the effects of miglitol on GLP1 secretion with those of acarbose, which failed to depolarize the *Xenopus laevis* oocytes expressing human SGLT3. Oral administration of miglitol activated duodenal enterochromaffin (EC) cells as assessed by immunostaining of phosphorylated calcium–calmodulin kinase 2 (phospho-CaMK2). In contrast, acarbose activated much fewer enteroendocrine cells, having only modest phospho-CaMK2 immunoreactivity. Single administration of miglitol triggered no GLP1 secretion, and GLP1 secretion by miglitol plus maltose was significantly attenuated by atropine pretreatment, suggesting regulation via vagal nerve. Thus, while α-GIs generally delay carbohydrate absorption and potentiate GLP1 secretion, miglitol also activates duodenal EC cells, possibly via SGLT3, and potentiates GLP1 secretion through the parasympathetic nervous system.

## Introduction

Oral ingestion of nutrients triggers the secretion of gut hormones from various enteroendocrine cells (Ezcurra *et al*. 2013, Cho *et al*. 2014). Among these, glucagon-like peptide 1 (GLP1) and glucose-dependent insulinotropic polypeptide (GIP) play a central role in dampening postprandial hyperglycemia after carbohydrate ingestion. Although both GLP1 and GIP are principal incretin hormones that increase insulin secretion, GLP1 is of special interest for its therapeutic potential in type 2 diabetes mellitus (T2DM; Cho *et al*. 2014). GLP1-targeted anti-diabetic agents comprise GLP1 mimetics and inhibitors of dipeptidyl peptidase 4 that enhance GLP1 action by inhibiting the rapid inactivation of the hormone endogenously secreted from enteroendocrine L cells. The mechanism of carbohydrate-induced GLP1 secretion is therefore critical in the physiology and pathophysiology of glucose homeostasis after meal ingestion. However, the molecular mechanism of carbohydrate-induced GLP1 secretion remains unclear.

Glucose is a potent stimulant of GLP1 secretion. As glucose is the most important energy source and its ambient levels need to be maintained, many cell types in the body are equipped with a glucose-sensing apparatus. These cells include pancreatic β-cells, glucose-responsive neurons in the hypothalamus, portal glucose sensors, and enteroendocrine cells. Pancreatic β-cells and glucose-responsive neurons are electrically excitable cells with a firing rate regulated by extracellular glucose levels. The molecular mechanisms of glucose sensing in these two cell types have been studied extensively. At present, several molecules such as glucose transporter 2 (GLUT2) (Thorens 2003), glucokinase (Matschinsky *et al*. 2006), and the ATP-sensitive K^+^ (K_ATP_) channel (Miki & Seino 2005) have been shown to be critical in glucose sensing in these cells. L cells are morphologically polarized, open-type endocrine cells. L cells are adjoined with enterocytes in gut mucosa and possess an apical neck with microvilli, in which nutrient in the gut lumen is recognized. While glucose is taken up into enterocytes through sodium-glucose co-transporter 1 (SGLT1) and diffuses into basolateral space through GLUT2, L cells also express both SGLT1 (SLC5A1) and GLUT2 (SLC2A2) (Reimann *et al*. 2008). In this study, we evaluated the physiological relevance of the K_ATP_ channel, SGLT1, and GLUT2 in the glucose sensing of L cells by measuring portal GLP1 concentrations *in vivo*.

During the course of the study, we noticed that the α-glucosidase inhibitor (α-GI) miglitol potentiates GLP1 secretion when co-administered with maltose. Inhibitors of α-glucosidase (glucan 1,4-alpha-glucosidase, Enzyme Commission (EC) number; 3.2.1.3) are generally considered to increase postprandial GLP1 levels through delaying digestion and absorption of carbohydrate (Seifarth *et al*. 1998, Enc *et al*. 2001). To investigate the mechanism of miglitol on GLP1 secretion, we examined its action on the putative glucose sensor SGLT3, which was originally identified in brain (Diez-Sampedro *et al*. 2003, Voss *et al*. 2007).

In this study, we compared two α-GIs, miglitol and acarbose, by examining their effects on GLP1 secretion, SGLT3-mediated depolarization in *Xenopus laevis* oocytes expressing human SGLT3 (hSGLT3), and enteroendocrine cell activation using immunostaining of phosphorylated calcium–calmodulin kinase 2 (phospho-CaMK2).

## Materials and methods

### Reagents

Phlorizin, phloretin, and acarbose were purchased from Wako Pure Chemical Industries, Ltd (Osaka, Japan). Miglitol was provided by Sanwa Kagaku Kenkyusho Co. Ltd (Nagoya, Aichi, Japan).

### Animal experiments

WT and *Kir6.2* (*Kcnj11*)-deficient male mice (Miki *et al*. 1998) with the same genetic background as that of C57BL/6 mice were used for the study. All animal studies were approved by the Animal Care and Use Committee of Chiba University.

### Experiments of GLP1 secretion *in vivo*

After 16-h fast, mice were subjected to GLP1 secretion experiments. We utilized two protocols: one for early-phase (5 min) and the other for late-phase (10 and 30 min) GLP1 secretion after sugar loading. For analyzing early-phase secretion, mice were anesthetized by pentobarbital and isoflurane and were laparotomized to place a gastric gavage in the duodenum. Five minutes after secretagogue (20 μl/g body weight) administration through gavage, portal blood (∼600 μl) was drawn and immediately mixed with EDTA (final 0.15% w/v) and diprotin A (final, 3 mmol/l). For analyzing late-phase (10 and 30 min) secretion, conscious mice were administered secretagogue (20 μl/g body weight). Under anesthesia with pentobarbital and isoflurane, portal blood (∼600 μl) was drawn in the same way as described earlier for early-phase sampling. The plasma intact GLP1 concentration was measured using Active GLP1 Assay Kit (Millipore, Billerica, MA, USA).

### Histological examination

Immunostaining of phospho-CaMK2 of gut was carried out under standardized methods. After administration of secretagogues (20 μl/g body weight), mice were killed by pentobarbital and isoflurane, perfused, and fixed with 4% paraformaldehyde. The duodenum was excised and tissues were embedded in O.C.T. Compound (Sakura Finetechnical Co., Tokyo, Japan). Frozen sections of 10 μm in thickness were incubated in 1% goat serum dissolved in PBS-T (PBS containing 0.3% Tween-20) for 30 min at room temperature. For double immunofluorescence of phospho-CaMK2 and 5-HT, sections were first stained with a rabbit anti-pCaMK2 (pT^286^) antibody (1:1000 in PBS-T, Promega) overnight at 4 °C. Incubation with the secondary antibody (goat anti-rabbit IgG antibody conjugated with AlexaFluor 488, 1:500 in PBS, Invitrogen Molecular Probes) was performed for 2 h at room temperature. Subsequently, the sections were incubated with a mouse anti-5-HT antibody (1:500 in PBS-T, Dako Corp., Carpenteria, CA, USA) overnight at 4 °C. The goat anti-mouse IgG antibody conjugated with AlexaFluor 594 (1:500 in PBS, Invitrogen Molecular Probes) was used thereafter as a secondary antibody. The slides were examined under a FluoView FV10i confocal microscope (Olympus, Tokyo, Japan).

### Electrophysiological recording of *X. laevis* oocytes expressing hSGLT3

As SGLT3 does not transport glucose but carries Na^+^ to elicit membrane depolarization (Diez-Sampedro *et al*. 2003), the function of SGLT3 was evaluated electrophysiologically in the *X. laevis* oocytes heterologously expressing human SGLT3. *hSGLT3* cDNA was inserted into pF1K T7 Flexi vector. The cDNA was linearized by FspI and used to synthesize cRNA using the mMESSAGE mMACHINE T7 RNA polymerase kit (Ambion, Austin, TX, USA). The polyadenylation of cRNA at 3′-end was performed using the Poly(A) tailing kit (Ambion). Small pieces of ovary were removed from *X. laevis* frogs. Follicles were isolated and treated with 1 mg/ml collagenase (Wako Pure Chemicals) in Ca^2+^-free solution (96 mmol/l NaCl, 2 mmol/l KCl, 1 mmol/l MgCl_2_, 5 mmol/l HEPES, pH 7.5) for 1.5–2 h at room temperature. Then, 50 nl of *hSGLT3* cRNA (0.5 μg/μl) was injected into defolliculated oocytes and maintained at 18 °C in ND96 buffer (96 mmol/l NaCl, 2 mmol/l KCl, 1 mmol/l CaCl_2_, 1 mmol/l MgCl_2_, 5 mmol/l HEPES, pH 7.5) supplemented with 2.5 mmol/l pyruvate and 50 μg/ml gentamicin. Three days after *hSGLT3* cRNA injection, the oocytes were used for electrophysiological recording. The oocytes were voltage clamped at a holding potential of −60 mV using two electrodes connected to an OC-725C amplifier (Warner Instruments, Hamden, CT, USA). To obtain an I–V relationship, the voltage was ramped to +60 mV (1 s duration) at intervals of 1 min.

### Statistical analysis

Results are expressed as means±s.e.m. Differences between two groups were assessed using unpaired two-tailed Student's *t*-test unless otherwise specified. Datasets involving more than two groups were assessed by one-way ANOVA. *P*<0.05 was considered statistically significant.

## Results

### Evaluation of involvement of SGLT1, GLUT2, and K_ATP_ channel in glucose-induced GLP1 secretion

**Figure 1:**
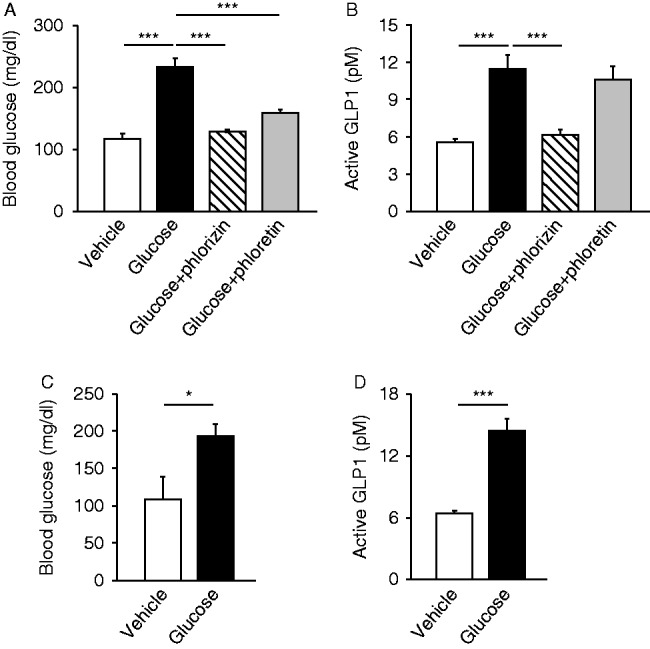
Physiological relevance of SGLT1, GLUT2, and K_ATP_ channel in glucose-induced GLP1 secretion in the early phase. Blood glucose (A) and plasma GLP1 concentrations (B) in portal vein at 5 min after intraduodenal administration of glucose (or vehicle) are shown. Glucose was co-administrated with or without phlorizin (500 mg/kg) or phloretin (500 mg/kg) to WT mice (A and B). Blood glucose (C) and plasma GLP1 concentrations (D) in portal vein of *Kir6.2*-deficient mice at 5 min after intraduodenal glucose administration are also shown. Data are expressed as mean±s.e.m. *P<0.05 and ***P<0.001.

We first examined GLP1 secretion *in vivo* by measuring blood glucose levels (Fig. 1A) and plasma GLP1 concentrations (Fig. 1B) in portal vein of anesthetized mice at 5 min after intraduodenal glucose (2 g/kg) administration, and found that the GLP1 concentrations were significantly increased (*n*=4–8, *P*<0.001). By contrast, when glucose was administered intraperitoneally, there was no increase in GLP1 concentration in spite of the significant rise in blood glucose level (data not shown), which accords with a previous report that glucose is recognized from the luminal side of the gut (Reimann *et al*. 2008, Cho *et al*. 2014).

We then evaluated the involvement of SGLT1 and GLUT2 in GLP1 secretion. Intraduodenal administration of the SGLT1 blocker phlorizin with glucose significantly suppressed glucose-induced GLP1 secretion (*n*=8, *P*<0.001) (Fig. 1B) along with inhibition of the rise in portal glucose levels (*P*<0.001) (Fig. 1A). By contrast, administration of the GLUT2 blocker phloretin together with glucose did not affect glucose-induced GLP1 secretion (*n*=4, not significant (NS)) (Fig. 1B), and the rise in portal glucose was significantly attenuated by phloretin (*P*<0.001) (Fig. 1A), indicating its effective blockade of glucose efflux from enterocytes into blood. These results suggest that glucose transport through SGLT1 into L cells is critical in GLP1 secretion under this experimental condition. We also assessed the involvement of the K_ATP_ channel in glucose-induced GLP1 secretion using K_ATP_ channel-deficient *Kir6.2* ^*−/−*^ mice. Intraduodenal glucose administration evoked a significant rise in blood glucose levels (*n*=5–8, *P*<0.05) and GLP1 secretion (*P*<0.001) in *Kir6.2* ^*−/−*^ mice (Fig. 1C and D), similar to that in WT mice, indicating that K_ATP_ channels are not essential for GLP secretion.

**Figure 2:**
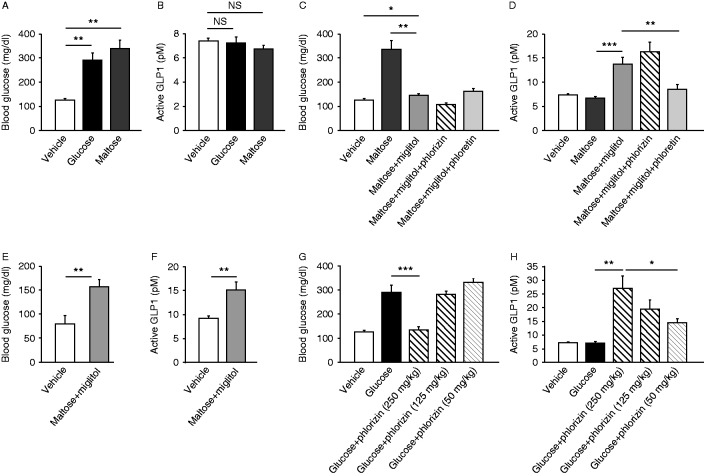
Physiological relevance of SGLT1 and GLUT2 in GLP1 secretion by maltose plus miglitol in the late phase. Blood glucose (A and C) and plasma GLP1 concentrations (B and D) in the portal vein of awake, WT mice at 30 min are shown. Data after oral administration of glucose, maltose, or vehicle (A, B, C, and D) or of maltose plus miglitol with or without either phlorizin or phloretin (C and D) are shown. Blood glucose (E) and plasma GLP1 concentrations (F) in portal vein of *Kir6.2* ^*−/−*^ mice at 30 min after oral administration of maltose plus miglitol are shown. Effect of different doses (250, 125, and 50 mg/kg) of phlorizin on glucose-induced GLP1 secretion is shown (G and H). Data are expressed as mean±s.e.m. *P<0.05, **P<0.01, and ***P<0.001.

To investigate the GLP1 secretory mechanism under more physiological conditions, we measured blood glucose levels and GLP1 secretion in unrestrained, conscious mice 30 min after oral administration of glucose (*n*=12, NS) or maltose (*n*=5, NS) (Fig. 2A and B). No significant increase in GLP1 concentrations by stimulation of either glucose or maltose was found, despite the fact that the portal glucose levels remained elevated at sampling. We then examined the GLP1 secretion at earlier time points (5 and 10 min) after oral glucose loading in mice under anesthesia with isoflurane (for 5 min) or under awake condition (for 10 min). The portal GLP1 levels were significantly increased at 5 min after oral glucose loading (15.93±1.80 pM, *n*=4, *P*<0.01). In addition, at 10 min after oral glucose loading, a significant increase in GLP1 concentrations was apparent (19.99±1.94 pM, *n*=7, *P*<0.001). These data suggest that the lack of increase at 30 min after oral glucose loading is probably due to the disappearance of residual luminal glucose at this time point.

We then co-administered the α-GI miglitol plus maltose and measured blood glucose levels and GLP1 concentrations at 30 min (Fig. 2C and D). In this protocol, the blood glucose levels were slightly but significantly increased by administration of miglitol plus maltose (*n*=5–10, *P*<0.05), but the rise was significantly smaller than that by maltose alone (*P*<0.01). By contrast, co-administration of miglitol significantly increased the GLP1 secretion by maltose (*P*<0.001). We then evaluated the involvement of SGLT1 and GLUT2 in GLP1 secretion induced by maltose plus miglitol in WT mice. Unlike GLP1 secretion by intraduodenal glucose, the GLP1 secretion by oral maltose plus miglitol was significantly suppressed by phloretin (*n*=6, *P*<0.01) but not by phlorizin (*n*=15, NS) (Fig. 2C), suggesting involvement of GLUT2 but not of SGLT1 in this condition. To evaluate involvement of K_ATP_ channels in GLP1 secretion in the late phase, we examined the rise in blood glucose levels and the GLP1 secretion by oral administration of maltose plus miglitol in *Kir6.2* ^*−/−*^ mice (Fig. 2E and F), and found that the GLP1 secretion also occurred in *Kir6.2* ^*−/−*^ mice (*n*=6–9, *P*<0.01).

### Effect of residual glucose in the gut lumen on GLP1 secretion

Hypothesizing that GLP1 secretion by maltose plus miglitol might be triggered by residual glucose in the gut lumen, we examined whether blockade of luminal glucose absorption by phlorizin restores the lack of secretion at 30 min. As expected, co-administration of phlorizin (250 mg/kg body weight) with glucose significantly suppressed the rise in blood glucose levels (*n*=10, *P*<0.001) and increased GLP1 secretion (*P*<0.01) (Fig. 2G and H). Interestingly, the relatively low dose of phlorizin (125 or 50 mg/kg body weight) did not suppress the rise in blood glucose levels after glucose load, but evoked significant GLP1 secretion (*n*=7, *P*<0.05 for 125 mg/kg phlorizin and *n*=13, *P*<0.001 for 50 mg/kg) in the dose-dependent manner of phlorizin.

### Comparison between miglitol and acarbose on GLP1 secretion induced by maltose

**Figure 3:**
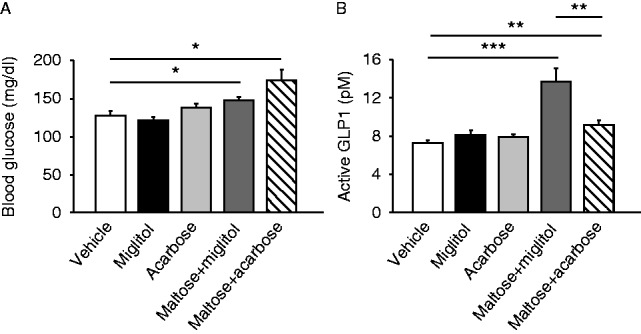
Comparison between miglitol and acarbose of GLP1 secretion induced by maltose. Blood glucose (A) and plasma GLP1 concentrations (B) in the portal vein at 30 min after oral administration of miglitol, acarbose, maltose plus miglitol, or maltose plus acarbose are shown. Data are expressed as mean±s.e.m. *P<0.05, **P<0.01 and ***P<0.001.

Then, we compared the effects of miglitol and acarbose on GLP1 secretion in mice. Single administration of neither miglitol nor acarbose affected blood glucose levels or GLP1 concentrations (*n*=14–16, NS) (Fig. 3A and B). By contrast, co-administration of miglitol (*P*<0.001) or acarbose (*P*<0.01) with maltose both increased GLP1 secretion (*n*=9–15). Miglitol plus maltose evoked significantly larger secretion than acarbose plus maltose (*n*=9–15, *P*<0.01), suggesting that miglitol may play a role in GLP1 secretion through a mechanism other than its α-GI activity.

### Electrophysiological recording of *X. laevis* oocytes expressing hSGLT3

In searching for a novel action of miglitol through a mechanism other than α-glucosidase inhibition, we investigated its effect on SGLT3, which has been reported to function as a glucose sensor in cholinergic neurons and skeletal muscles (Diez-Sampedro *et al*. 2003). SGLT3 has also been shown to be expressed in the small intestine (Barcelona *et al*. 2012). However, the expression of mouse SGLT3 (SGLT3a (SLC5A4A) and SGLT3b (SLC5A4B)) in the sub-regions of small intestine has not been examined. We therefore evaluated *Sglt3a* and *Sglt3b* mRNA expressions in duodenum, jejunum, and ileum by RT-PCR in mice (data not shown). Although expression of SGLT3a and SGLT3b was identified in all sub-regions, the expression levels were relatively low in ileum, in which L cells exist most abundantly.

**Figure 4:**
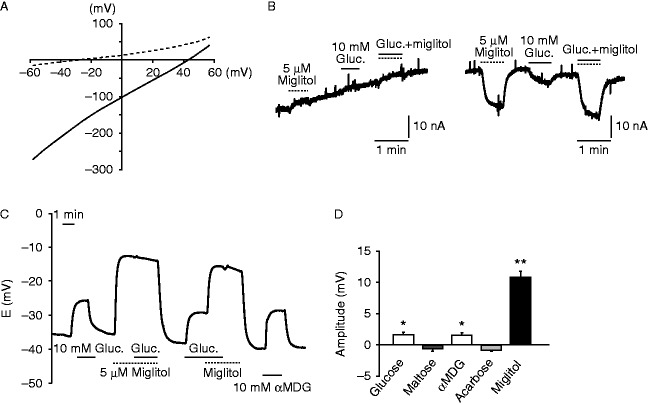
Electrophysiological recording of *Xenopus laevis* oocytes expressing hSGLT3. (A, B, C, and D) (A) I–V relationship of the oocytes expressing hSGLT3 in the presence (solid line) or absence (dashed line) of miglitol. (B) A representative recording of whole-cell current under voltage clamp mode in an oocyte expressing hSGLT3 in the absence (left) or presence (right) of Na^+^ in the buffer. ‘Gluc.’ denotes glucose. (C) A representative recording of the membrane potentials under current clamp mode in an oocyte expressing hSGLT3 in response to glucose and/or miglitol. The SGLT3 agonist α-methyl-d-glucopyranoside (αMDG) was used as a positive control for SGLT3-dependent depolarization. ‘Gluc.’ denotes glucose. (E) The current changes by several stimuli (*n*=4 for each). Data are expressed as mean±s.e.m. *P<0.05 and **P<0.01 (compared with the base line).

Miglitol shares α-glucosidase inhibitory properties with other α-GIs such as voglibose and acarbose. As miglitol and acarbose differ structurally (Gloster & Vocadlo 2012), we expected that acarbose does not activate SGLT3. To ascertain this, we examined the electrophysiological effect of miglitol on SGLT3 using *X. laevis* oocytes expressing hSGLT3. As reported previously (Barcelona *et al*. 2012), miglitol depolarized the cells in a Na^+^-dependent manner (Fig. 4A and B). Administration of glucose or miglitol elicited a sharp and reversible depolarization of hSGLT3-expressing oocytes (Fig. 4C). In our protocols, additive or synergic effects of glucose and miglitol were not observed. In contrast, acarbose failed to depolarize the membrane (*n*=4 for each group) (Fig. 4D). We therefore compared the effects of miglitol and acarbose on enteroendocrine cell activation.

### Histological evaluation of CaMK2 activation in duodenal enteroendocrine cells by glucose, maltose plus miglitol, miglitol, or acarbose

**Figure 5:**
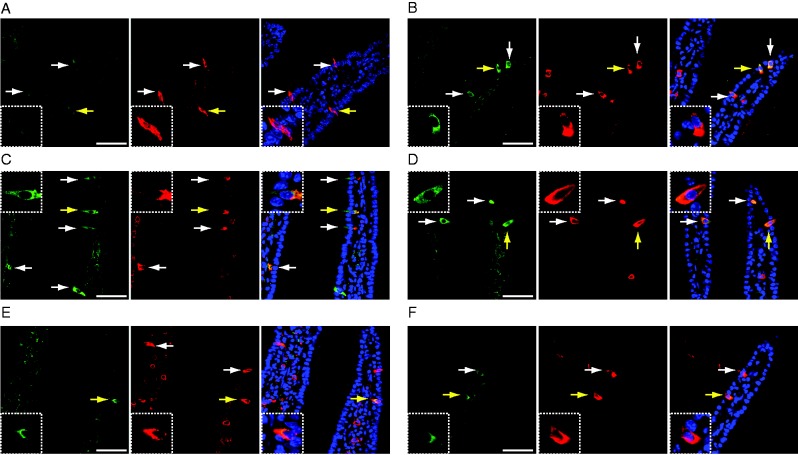
Activation of CaMK2 of duodenal enteroendocrine cells. (A, B, C, D, and E) Immunohistochemistry of phospho-CaMK2 (green) and 5-HT (red) of duodenum is shown. (A) Oral vehicle administration. (B) Intraduodenal glucose administration. (C) Oral maltose plus miglitol administration. (D) Oral miglitol administration. (E) Oral acarbose administration. (F) Intraperitoneal glucose administration. Insets denote the cells indicated by yellow arrows at a higher magnification. White arrows indicate immunopositive cells. (A, B, C, D, and E) Images of phospho-CaMK2 (left), 5-HT (middle), and their superposition (phospho-CaMK2, 5-HT, and DAPI). Bars indicate 50 μm.

To evaluate cellular activation in the gut, we examined phosho-CaMK2-positive cells in the duodenum from mice administered (orally, intraperitoneally, or intraduodenally) glucose, maltose plus miglitol, miglitol, acarbose, or vehicle (Fig. 5A, B, C, D, E, and F). As reported previously (Vincent *et al*. 2011), intraduodenal glucose increased the number of phospho-CaMK2-positive cells (Fig. 5B), but oral vehicle (Fig. 5A) or i.p. glucose administration had no effect (Fig. 5F). The signals of AlexaFluor 594 (conjugated with a secondary antibody against 5-HT) in the cells located inside of intestinal villi are non-specific, as this was similarly detected in the immunostaining without a primary antibody (mouse anti-5-HT antibody) (Supplementary Figure 1, see section on supplementary data given at the end of this article, see details in supplementary data given at the end of the article).

Co-immunostaining of 5-HT and phospho-CaMK2 revealed that most phospho-CaMK2-positive enteroendocrine cells were positive for 5-HT, indicating that these cells were enterochromaffin (EC) cells. In contrast, phosphorylation of CaMK2 was considered not to occur in enteroendocrine L cells either in duodenum or in ileum, as most phospho-CaMK2-positive enteroendocrine cells are EC cells, which constitute a distinct cell population from L cells (Wang *et al*. 2004).

In addition, oral administration of maltose plus miglitol also evoked phosphorylation of CaMK2 (Fig. 5C). Considering the parallels of CaMK2 phosphorylation in EC cells and GLP1 secretion from L cells by various stimuli, we then examined the effects of single administration of either miglitol or acarbose on CaMK2 phosphorylation. Oral administration of miglitol activated duodenal enteroendocrine cells (Fig. 5D). However, in contrast to miglitol, administration of acarbose induced CaMK2 phosphorylation in a lesser number of enteroendocrine cells, with a modest immunoreactivity, to a level comparable to that in mice administered vehicle alone (Fig. 5E).

### Evaluation of involvement of vagal nerves in GLP1 secretion by oral administration of maltose plus miglitol

**Figure 6:**
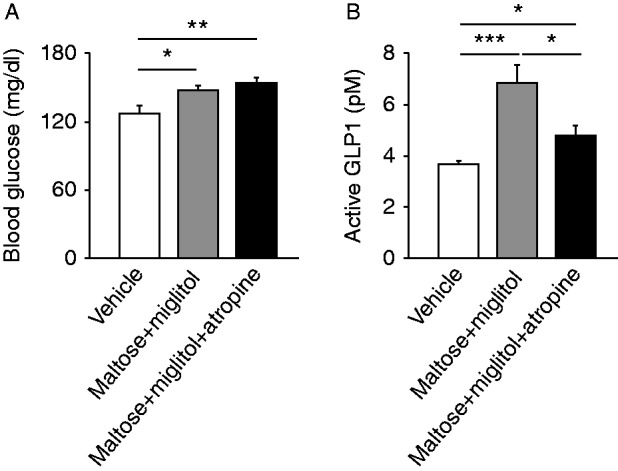
Involvement of parasympathetic nerves in GLP1 secretion. Blood glucose (A) and plasma GLP1 concentrations (B) in the portal vein at 30 min after oral administration of maltose plus miglitol with or without atropine pretreatment are shown. Data are expressed as mean±s.e.m. *P<0.05, **P<0.01, and ***P<0.001.

To clarify autonomic nerve involvement, we pretreated mice with a muscarinic receptor antagonist, atropine, and measured blood glucose levels and GLP1 concentrations (Fig. 6A and B). GLP1 secretion by miglitol plus maltose was significantly attenuated by atropine pretreatment (*n*=6–10, *P*<0.05), indicating parasympathetic nervous system involvement in GLP1 secretion.

## Discussion

GLP1 is secreted from enteroendocrine L cells in response to oral nutrient ingestion. Carbohydrates, peptides, and lipids in ingested meal are all known to trigger GLP1 secretion (Cho *et al*. 2014, Ohlsson *et al*. 2014). Glucose is a potent secretagogue of GLP1 secretion. Recent studies of primary L cells and the L cell line GLUTag have clarified that L cells express various genes encoding putative glucose sensors, including *Sglt1*, *Glut2*, K_ATP_ channel subunits (*Kir6.2* and *Sur1* (*Abcc8*)), glucokinase, and sweet receptor-related genes (*Tas1r2*/*Tas1r3* and α-gastducin) (Cho *et al*. 2014). However, whether or not SGLT1 and GLUT2 are involved in GLP1 secretion from L cells remains controversial. Some studies have found an essential role of SGLT1 (Reimann *et al*. 2008, Moriya *et al*. 2009, Gorboulev *et al*. 2012); others have emphasized the importance of GLUT2 (Cani *et al*. 2007, Mace *et al*. 2012). In this study, we found intraduodenal glucose administration to elicit early-phase (5 min) GLP1 secretion, which was blocked by phlorizin (Fig. 1B), while delayed-phase (30 min) GLP1 secretion by maltose plus miglitol was inhibited by phloretin (Fig. 2D). Interestingly, Powell *et al*. (2013) reported that GLP1 secretion in *Sglt1*-deficient mice was significantly lower at 5 min but much higher at 1 h and later after glucose loading compared with that in WT mice, suggesting a complex contribution of SGLT1 depending on the time course (Powell *et al*. 2013). Considered together with our present data, we suggest that SGLT1 may be critical for the early phase of glucose-induced GLP1 secretion and that GLUT2 is involved in the late phase, although it remains unknown whether SGLT1 or GLUT2 expressed in L cells participates in this secretion. Regarding the role of K_ATP_ channels, several papers have reported that K_ATP_ channels in L cells play an important role in GLP1 secretion (Reimann *et al*. 2008, Mace *et al*. 2012). However, our results include direct evidence that K_ATP_ channels are not essential in GLP1 secretion, although this does not imply that the channels have no role in its regulation.

Co-administration of phlorizin with glucose dose dependently increased GLP1 secretion (Fig. 2H), suggesting that the existence of glucose in the gut lumen is important for triggering the GLP1 secretion in the late phase after glucose load and that this secretion is mediated in a SGLT1-independent manner. This concept is compatible with the clinical observation that α-GIs, which delay the hydrolysis of di-, tri-, and polysaccharides, generally potentiate carbohydrate-induced GLP1 secretion (Hiki *et al*. 2010, Sakurai *et al*. 2012). In addition, miglitol was reported to potentiate GLP1 secretion more robustly than acarbose, despite a comparable glucose-lowering effect after meal ingestion (Arakawa *et al*. 2008). We therefore considered that the effect of miglitol on GLP1 secretion is unlikely to be mediated only by its α-GI activity. GLP1 secretion by miglitol plus maltose was larger than that by acarbose plus maltose (Fig. 3B), despite the fact that we applied a much higher dose of acarbose in terms of enzymatic inhibition of maltase (Samulitis *et al*. 1987). Accordingly, miglitol would seem to retain a potentiating effect on GLP1 secretion through a mechanism other than α-glucosidase inhibition.

In searching for a novel mechanism of miglitol action, we evaluated its effect on SGLT3 (Diez-Sampedro *et al*. 2003). We found that SGLT3 is expressed in all sub-regions of small intestine and to a lesser extent in ileum. Similar to SGLT1, SGLT3 is activated by glucose and elicits Na^+^-dependent depolarization. However, SGLT3 does not transport glucose (Barcelona *et al*. 2012) and is therefore considered to be a glucose sensor. SGLT3 has been shown to be activated by several imino-sugars, including 1-deoxynojirimycin (DNJ), *N*-butyl-1-deoxynojirimycin (miglustat), and *N*-hydroxyethyl-a-deoxynojirimycin (miglitol) (Diez-Sampedro *et al*. 2003, Barcelona *et al*. 2012).

Miglitol and acarbose are structurally different (Gloster & Vocadlo 2012) and our electrophysiological recordings revealed that miglitol and glucose but not acarbose activate hSGLT3 (Fig. 4D), which led us to compare the effects of miglitol and acarbose on enteroendocrine cell activation. Notably, administration of glucose (intraduodenally) or miglitol (orally) evoked CaMK2 phosphorylation, but acarbose failed to elicit CaMK2 phosphorylation (Fig. 5). Although a single administration of miglitol activated enteroendocrine cells in the duodenum, it failed to increase the GLP1 secretion (Fig. 3B). Miglitol is therefore unlikely to act directly on L cells to trigger the GLP1 secretion.

Nutrient sensing in gut lumen has been reported to play important roles in glucose homeostasis. Especially, jejunum and duodenum are known to play an important role in glucose sensing to regulate glucose production in the liver (Breen *et al*. 2012, 2013, Marina *et al*. 2012). Considering that L cells exist abundantly in the terminal ileum (Tolhurst *et al*. 2009), there might well be a mechanism linking nutrient sensing in the upper small intestine with GLP1 secretion in the ileum. In addition, our results suggest that the parasympathetic nervous system is probably involved in GLP1 secretion (Fig. 6A and B). This accords with the previous report that postprandial GLP1 secretion is mediated through the vagal nervous system in rats (Anini *et al*. 2002).

Accordingly, we propose a model for GLP1 secretion by maltose plus miglitol as follows: miglitol in the luminal side of the duodenum activates 5-HT-positive EC cells via SGLT3 to trigger the secretion of 5-HT, which activates the parasympathetic nervous system. Simultaneously, miglitol inhibits the hydrolysis of maltose, leading to the increase in the amount of glucose in the ileum, in which L cells exist abundantly. The combination of the existence of luminal glucose in the ileum and parasympathetic nervous input to L cells then evokes GLP1 secretion at late phase (30 min) after oral administration.

Considering the efficacy of GLP1-targeted therapy in the treatment of T2DM, augmentation of GLP1 secretion may be a novel therapeutic target of the disease.

## Supplementary data

This is linked to the online version of the paper at http://dx.doi.org/10.1530/JOE-14-0555.

Supplementary Figure

## References

[bib1] Anini Y, Hansotia T, Brubaker PL (2002). Muscarinic receptors control postprandial release of glucagon-like peptide-1: *in vivo* and *in vitro* studies in rats. Endocrinology.

[bib2] Arakawa M, Ebato C, Mita T, Fujitani Y, Shimizu T, Watada H, Kawamori R, Hirose T (2008). Miglitol suppresses the postprandial increase in interleukin 6 and enhances active glucagon-like peptide 1 secretion in viscerally obese subjects. Metabolism.

[bib3] Barcelona S, Menegaz D, Diez-Sampedro A (2012). Mouse SGLT3a generates proton-activated currents but does not transport sugar. American Journal of Physiology. Cell Physiology.

[bib4] Breen DM, Rasmussen BA, Kokorovic A, Wang R, Cheung GW, Lam TK (2012). Jejunal nutrient sensing is required for duodenal–jejunal bypass surgery to rapidly lower glucose concentrations in uncontrolled diabetes. Nature Medicine.

[bib5] Breen DM, Rasmussen BA, Cote CD, Jackson VM, Lam TK (2013). Nutrient-sensing mechanisms in the gut as therapeutic targets for diabetes. Diabetes.

[bib6] Cani PD, Holst JJ, Drucker DJ, Delzenne NM, Thorens B, Burcelin R, Knauf C (2007). GLUT2 and the incretin receptors are involved in glucose-induced incretin secretion. Molecular and Cellular Endocrinology.

[bib7] Cho YM, Fujita Y, Kieffer TJ (2014). Glucagon-like peptide-1: glucose homeostasis and beyond. Annual Review of Physiology.

[bib8] Diez-Sampedro A, Hirayama BA, Osswald C, Gorboulev V, Baumgarten K, Volk C, Wright EM, Koepsell H (2003). A glucose sensor hiding in a family of transporters. PNAS.

[bib9] Enc FY, Imeryuz N, Akin L, Turoglu T, Dede F, Haklar G, Tekesin N, Bekiroglu N, Yegen BC, Rehfeld JF (2001). Inhibition of gastric emptying by acarbose is correlated with GLP-1 response and accompanied by CCK release. American Journal of Physiology. Gastrointestinal and Liver Physiology.

[bib10] Ezcurra M, Reimann F, Gribble FM, Emery E (2013). Molecular mechanisms of incretin hormone secretion. Current Opinion in Pharmacology.

[bib11] Gloster TM, Vocadlo DJ (2012). Developing inhibitors of glycan processing enzymes as tools for enabling glycobiology. Nature Chemical Biology.

[bib12] Gorboulev V, Schurmann A, Vallon V, Kipp H, Jaschke A, Klessen D, Friedrich A, Scherneck S, Rieg T, Cunard R (2012). Na(+)-d-glucose cotransporter SGLT1 is pivotal for intestinal glucose absorption and glucose-dependent incretin secretion. Diabetes.

[bib13] Hiki M, Shimada K, Kiyanagi T, Fukao K, Hirose K, Ohsaka H, Fukushima Y, Kume A, Matsumori R, Sumiyoshi K (2010). Single administration of α-glucosidase inhibitors on endothelial function and incretin secretion in diabetic patients with coronary artery disease. Circulation Journal.

[bib14] Mace OJ, Schindler M, Patel S (2012). The regulation of K- and L cell activity by GLUT2 and the calcium-sensing receptor CasR in rat small intestine. Journal of Physiology.

[bib15] Marina AL, Utzschneider KM, Wright LA, Montgomery BK, Marcovina SM, Kahn SE (2012). Colesevelam improves oral but not intravenous glucose tolerance by a mechanism independent of insulin sensitivity and β-cell function. Diabetes Care.

[bib16] Matschinsky FM, Magnuson MA, Zelent D, Jetton TL, Doliba N, Han Y, Taub R, Grimsby J (2006). The network of glucokinase-expressing cells in glucose homeostasis and the potential of glucokinase activators for diabetes therapy. Diabetes.

[bib17] Miki T, Seino S (2005). Roles of K_ATP_ channels as metabolic sensors in acute metabolic changes. Journal of Molecular and Cellular Cardiology.

[bib18] Miki T, Nagashima K, Tashiro F, Kotake K, Yoshitomi H, Tamamoto A, Gonoi T, Iwanaga T, Miyazaki J, Seino S (1998). Defective insulin secretion and enhanced insulin action in K_ATP_ channel-deficient mice. PNAS.

[bib19] Moriya R, Shirakura T, Ito J, Mashiko S, Seo T (2009). Activation of sodium-glucose cotransporter 1 ameliorates hyperglycemia by mediating incretin secretion in mice. American Journal of Physiology. Endocrinology and Metabolism.

[bib20] Ohlsson L, Kohan AB, Tso P, Ahrén B (2014). GLP-1 released to the mesenteric lymph duct in mice: effects of glucose and fat. Regulatory Peptides.

[bib21] Powell DR, Smith M, Greer J, Harris A, Zhao S, DaCosta C, Mseeh F, Shadoan MK, Sands A, Zambrowicz B (2013). LX4211 increases serum glucagon-like peptide 1 and peptide YY levels by reducing sodium/glucose cotransporter 1 (SGLT1)-mediated absorption of intestinal glucose. Journal of Pharmacology and Experimental Therapeutics.

[bib22] Reimann F, Habib AM, Tolhurst G, Parker HE, Rogers GJ, Gribble FM (2008). Glucose sensing in L cells: a primary cell study. Cell Metabolism.

[bib23] Sakurai K, Lee EY, Morita A, Kimura S, Kawamura H, Kasamatsu A, Shiiba M, Yabe D, Yokote K, Miki T (2012). Glucagon-like peptide-1 secretion by direct stimulation of L cells with luminal sugar vs non-nutritive sweetener. Journal of Diabetes Investigation.

[bib24] Samulitis BK, Goda T, Lee SM, Koldovsky O (1987). Inhibitory mechanism of acarbose and 1-deoxynojirimycin derivatives on carbohydrases in rat small intestine. Drugs Under Experimental and Clinical Research.

[bib25] Seifarth C, Bergmann J, Holst JJ, Ritzel R, Schmiegel W, Nauck MA (1998). Prolonged and enhanced secretion of glucagon-like peptide 1 (7–36 amide) after oral sucrose due to α-glucosidase inhibition (acarbose) in type 2 diabetic patients. Diabetic Medicine.

[bib26] Thorens B (2003). A gene knockout approach in mice to identify glucose sensors controlling glucose homeostasis. Pflügers Archiv.

[bib27] Tolhurst G, Reimann F, Gribble FM (2009). Nutritional regulation of glucagon-like peptide-1 secretion. Journal of Physiology.

[bib28] Vincent KM, Sharp JW, Raybould HE (2011). Intestinal glucose-induced calcium-calmodulin kinase signaling in the gut-brain axis in awake rats. Neurogastroenterology and Motility.

[bib29] Voss AA, Diez-Sampedro A, Hirayama BA, Loo DD, Wright EM (2007). Imino sugars are potent agonists of the human glucose sensor SGLT3. Molecular Pharmacology.

