# Supplementary material for: Distinct action of the α-glucosidase inhibitor miglitol on SGLT3, enteroendocrine cells, and GLP1 secretion
Source: J Endocrinol. 2014 Dec 8;224(3):205–14. doi: 10.1530/JOE-14-0555 (PMC4324305; doi:10.1530/JOE-14-0555)
Supplement: Supplementary Figure [file supp_224_3_205__index.html]

Supplementary Figure 

# Distinct action of the α-glucosidase inhibitor miglitol on SGLT3, enteroendocrine cells, and GLP1 secretion

## Supplementary Figure

**Files in this Data Supplement:**

- Supplementary Figure 1 - (PDF 137 KB)
