# Supplementary figures and images for: Distinct action of the α-glucosidase inhibitor miglitol on SGLT3, enteroendocrine cells, and GLP1 secretion
Source: J Endocrinol. 2014 Dec 8;224(3):205–14. doi: 10.1530/JOE-14-0555 (PMC4324305; doi:10.1530/JOE-14-0555)

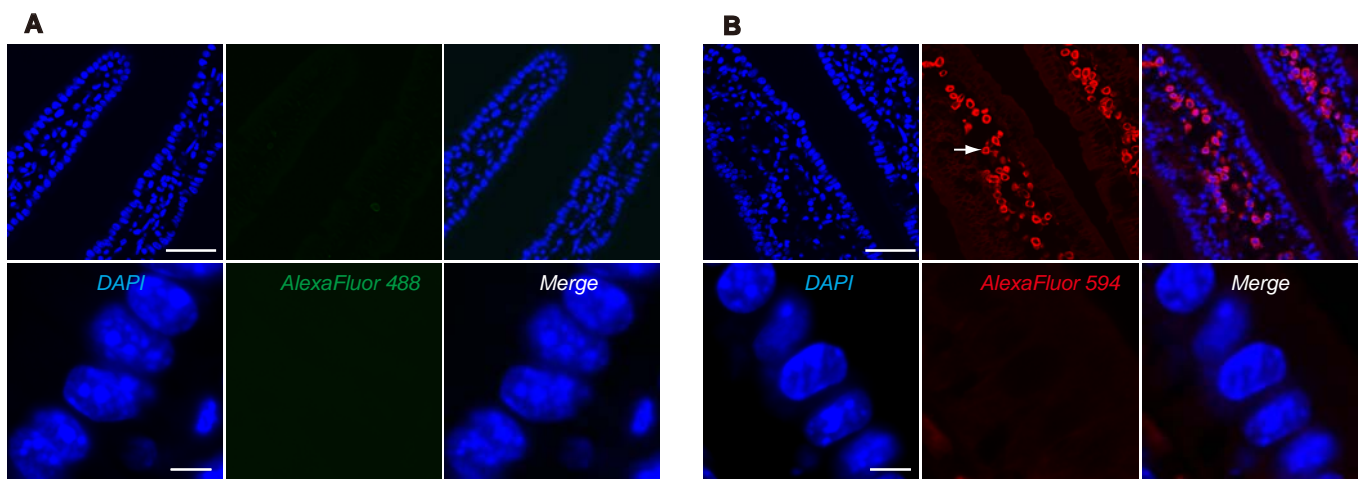

Supplement: Supplementary Figure [file supp_JOE-14-0555_Supplementary_figure_1.pdf]
